# Incidence of Notifiable Disease

**Published:** 2013-06

**Table:** (No of cases) in 2012*

Diseases	JAN	FEB	MAR	APR	MAY	JUN	JUL	AUG	SEP	OCT	NOV	DEC	Total
Diseases	No	No	No	No	No	No	No	No	No	No	No	No	No
AFP COMPATIBLE	0	0	0	0	0	0	0	0	0	0	0	0	0
AFP DISCARDED	55	59	17	50	64	62	25	51	41	25	35	41	525
AFP PENDING	5	8	16	6	8	2	16	5	2	13	17	17	115
AFP total	60	67	33	56	72	64	41	56	43	38	52	58	640
AFP VDPV	0	0	0	0	0	0	0	1	0	0	0	0	1
AIDS CUMULATIVE			3455			3619			3746			4097	4097
ANTHRAX	5	3	0	4	5	26	35	32	47	33	11	6	207
BRUCELLOSIS	903	819	871	1025	1506	1750	1970	1856	1519	1216	1064	907	15406
CCHF conf	0	0	1	8	27	28	8	10	4	2	2	2	92
CCHF Death	0	0	0	2	5	1	1	1	0	1	1	0	12
CCHF prob	2	9	4	18	73	74	32	27	17	10	7	11	284
CHOLERA	0	0	0	1	0	0	2	1	3	45	1	0	53
DIPHTHERIA-prob	14	14	8	10	17	14	15	17	6	16	14	11	156
GONOCOCCAL INFECTION	334	319	275	256	302	317	171	131	204	90	181	198	2778
HEPATITIS B	836	851	778	416	683	814	395	877	763	975	703	811	8902
HEPATITIS B&C	8	10	20	4	6	3	7	10	10	10	7	9	104
HEPATITIS B&D	0	0	0	0	0	0	0	0	0	0	0	0	0
HEPATITIS C	185	201	256	109	150	163	101	212	227	263	132	156	2155
HIV+ CUMULATIVE			24290			24735			25041			25573	25573
HYDATIC CYST	49	47	67	33	42	45	38	63	53	56	53	45	591
LEISHMANIASIS CUTANEUS	2304	1778	1172	816	1362	794	717	999	1485	2130	2495	2072	18124
LEISHMANIASIS VISCERAL	5	6	4	18	9	3	13	1	6	4	8	2	79
LEPROSY	0	1	1	3	3	1	2	2	3	1	1	3	21
LEPTOSPIROSIS	0	1	1	2	22	32	13	32	15	9	5	1	133
MALARIA	12	9	17	32	18	63	58	11	36	17	33	13	319
MEASLES conf	2	3	17	30	53	16	25	14	16	11	14	27	228
MEASLES susp	268	233	198	300	561	411	426	264	283	315	282	380	3921
MENINGOCOCCAL MENINGITIS	2	2	1	3	1	0	2	1	0	0	0	1	13
NEONATAL TETANUS	0	0	0	0	1	0	0	0	0	1	0	0	2
OTHER MENINGITIS	107	97	101	149	186	135	138	158	147	150	158	148	1674
POLIO	0	0	0	0	0	0	0	0	0	0	0	0	0
PULMONARY TB			2079			1883			2049			1746	7757
RABIES	0		0	2	0	0	0	0	0	0	0	3	5
RELAPSING FEVER	1	0	0	0	1	1	1	1	0	0	1	0	6
SYPHILIS	2	2	2	2	5	5	4	2	2	0	2	5	33
TETANUS	1	0	1	1	3	0	1	1	3	2	0	0	13
TUBERCULOSIS			2887			2701			2804			2358	10750
TYPHOID & PARATYPHOID	44	22	40	21	59	53	64	71	32	28	28	27	489
WHOOPING COUGH- susp	127	107	83	122	139	161	129	91	74	80	79	95	1287

* Source: Iranian Center for Disease Control

